# Associations of micronutrients and lipids with prediabetes and glycemic parameters in adolescent girls of the rural DERVAN cohort (DERVAN-9)

**DOI:** 10.3389/fnut.2024.1380777

**Published:** 2024-07-11

**Authors:** Suvarna Patil, Omkar Dervankar, Pallavi Hardikar-Bhat, Charudatta Joglekar, Rohit Bhat, Netaji Patil, Arvind Yadav

**Affiliations:** ^1^Department of Medicine, BKL Walawalkar Hospital and Rural Medical College, Ratnagiri, Maharashtra, India; ^2^Regional Centre for Adolescent Health and Nutrition, BKL Walawalkar Hospital and Rural Medical College, Ratnagiri, Maharashtra, India; ^3^Department of Radiology, BKL Walawalkar Hospital and Rural Medical College, Ratnagiri, Maharashtra, India; ^4^Department of Biochemistry, BKL Walawalkar Rural Medical College, Ratnagiri, Maharashtra, India

**Keywords:** micronutrients, lipids, prediabetes, undernutrition, India, adolescents, rural

## Abstract

**Background:**

We investigated the associations of micronutrients and lipids with prediabetes, glycemic parameters, and glycemic indices among the adolescent girls of the DERVAN (aDolescent and prEconception health peRspectiVe of Adult Non-communicable diseases) cohort study from rural India.

**Methods:**

We recruited 1,520 adolescent girls aged 16–18 years. We measured glycemic parameters (glucose, insulin and HbA_1_C), lipids (total cholesterol, high-density lipoprotein [HDL], low-density lipoprotein [LDL], and triglycerides), and micronutrients (vitamin B_12_, folate, and vitamin D). Prediabetes was defined using American Diabetes Association criteria (fasting glucose ≥100 mg/dL or HbA1C ≥5.7%). Glycemic indices (insulin resistance, insulin sensitivity, and β cell function) were calculated using the homeostasis model. Associations of prediabetes, glycemic parameters and glycemic indices with micronutrients and lipids were analyzed by multiple logistic regressions.

**Results:**

The median age and Body Mass Index (BMI) were 16.6 years and 17.6 kg/m^2^, respectively. Overall, 58% of girls had a low BMI. Median vitamin B_12_, folate, and vitamin D concentrations were 249.0 pg/mL, 6.1 ng/mL, and 14.2 ng/mL, respectively. The deficiencies observed were 32.1% for vitamin B_12_, 11.8% for folate, and 33.0% for vitamin D. Median total cholesterol, LDL, HDL, and triglyceride concentrations were 148.0 mg/dL, 81.5 mg/dL, 50.8 mg/dL, and 61.5 mg/dL, respectively. Elevated total cholesterol, LDL, and triglycerides were observed in 4.8, 4.0, and 3.8%, respectively, while low HDL was observed in 12.8%. Prediabetes was observed in 39.7% of the girls. Among lipids, total cholesterol and LDL were higher in girls with prediabetes (*p* < 0.01 for both). In a multivariate model containing cholesterol and vitamin B_12_/folate/vitamin D, prediabetes was associated with high cholesterol. Prediabetes was also associated with high LDL, independent of folate and vitamin D. Poor insulin secretion was high in those with low vitamin B_12_. Elevated insulin resistance was associated with low HDL. The likelihood of high insulin sensitivity was reduced in those with high triglycerides. The likelihood of poor β cell function was high in those with high LDL. Statistical interactions between micronutrients and lipids for prediabetes and glycemic outcomes were not significant.

**Conclusion:**

There was a substantial deficiency of micronutrients and an absence of dyslipidemia. Our results indicate the need for lipid and micronutrient-based interventions in adolescence to improve glycemic outcomes. Maintaining adequate storage of not only micronutrients but also lipids in adolescent girls is likely to reduce diabetes risk in adulthood.

## Introduction

Non-communicable diseases (NCDs) like diabetes and hypertension are on the rise in India (1). At the same time, the burden of cardiovascular diseases (CVD) is also increasing (2). An unhealthy diet, sedentary lifestyle, and substance abuse (smoking, tobacco chewing, and alcohol consumption) have been identified as major contributing factors. The rise in NCDs is very evident in urban India. Over the last few years, many rural communities have also started witnessing a rise, not only in CVD and coronary heart disease (CHD) (3, 4) but also in NCDs (5, 6). South Asians develop CHD earlier than white Caucasians (7). Lipid abnormalities have been identified as major risk factors for CVD as well as CHD (8, 9). The INTERHEART study (10) identified elevated levels of total cholesterol (CHOL) and low-density lipoprotein cholesterol (LDL) as risk factors for CHD in South Asians. Studies from India have shown increased CHOL levels not only in urban subjects but also in rural subjects (4, 11). According to the Developmental Origins of Health and Disease (DOHaD) hypothesis, seeds of CHD, CVD, and NCDs are sown in early life, covering the intrauterine period as well as early childhood and adolescence (12–14). There are reports from Europe and China on early life undernutrition leading to dyslipidemia (15–17) as well as diabetes (18, 19) in adult life. Though dyslipidemia has been interconnected to the pathophysiology of CVD, it is also a modifiable dominant risk factor if detected early in life (20). Dyslipidemia is uncommon in adolescence, but if it exists then it is expected to intensify the risk of CVD in adulthood (21). Lipid levels in adolescence are known to strongly correlate with those in later life (22, 23). High levels of LDL and low levels of high-density lipoprotein cholesterol (HDL) in adolescence are precursors for atherosclerosis in adulthood (24). There are very few studies regarding undernutrition and dyslipidemia in Indian adolescents (25–27). Between the years 2016–18, the Indian government carried out a Comprehensive National Nutrition Survey (CNNS) of adolescents aged 10–19 years, covering the entire nation with a sample size of >35,000 (28). It found increased lipid burden (21) as well as prediabetes (29). Over the last decade, the role of undernutrition in the development of diabetes has also been under investigation (30–33). Also, there are many reports investigating the role of micronutrients, which are biomarkers of nutrition, in the development of NCDs, especially diabetes (34–36).

BKL Walawalkar Hospital was established in the year 1996 in the village of DERVAN situated in the coastal region of Konkan in the western Indian state of Maharashtra. Since its inception, women’s health has been a prime area of interest. The hospital runs various holistic programs encompassing newborns, children, adolescent girls, newly married girls, and pregnant women. Comprehensive health education is provided, and various investigations are carried out. Counseling, holistic education, and medical treatment, if needed, are provided free of charge. Various hospital- and community-based cross-sectional, as well as observational, studies have demonstrated the presence of undernutrition across the entire life cycle (37–39). In addition, this region has also observed a rising prevalence of NCDs (diabetes and hypertension) (40). We have also demonstrated a high incidence of gestational diabetes among undernourished women in our region (41). This suggests an intergenerational link between an undernourished mother and her offspring.

The DERVAN cohort, a prospective longitudinal study of adolescent girls from the region, was set up in 2019 (42). Its objective was to test the hypothesis that poor physical growth and poor nutrition in adolescent girls increase the risk of NCDs, in particular the risk of diabetes in adulthood and in their offspring. Adolescent girls (16–18 years of age) were recruited between June 2019 and February 2023. The study is expected to continue for the next 20 years with an annual follow-up of adolescent girls.

Our recent report on this cohort showed a high prevalence of prediabetes (PD) among adolescent girls (43). Prediabetes precedes type 2 diabetes (T2D) and is marked by glucose levels above normal but below the diabetic threshold.

Baseline measurements of micronutrients, which are biomarkers of nutrition, as well as those of lipids, provided us the opportunity to investigate the role any of them may have in the development of PD in adolescence and diabetes in later life.

## Methods

The detailed protocol of the study is already reported (42). In short, 16–18-year-old adolescent girls born in Konkan, staying with parents with no history of any major illness (e.g., heart, kidney, liver disease, cancer, and psychiatric disorders), as well as with no history of mental, intellectual, or physical disability, were recruited. We recruited 1,520 girls at baseline. They were brought to the institute in groups of 5–7. They had an overnight stay at the hostel to ensure their fasting status. The detailed protocol for blood collection has already been reported (44).

Anthropometric measurements of height, weight, waist circumference, and hip circumference were made using a standardized protocol. Body fat was measured using a bioimpedance analyzer (MC-780, TANITA Corporation, Japan).

### Laboratory methods

A fasting blood sample was drawn from adolescent girls and further processed to measure micronutrients, lipids, and glycemic parameters. Blood samples were centrifuged (4°C, 3,000 rpm, 15 min) within 1 h of collection and stored at −80°C for further investigations.

Blood glucose was measured on the ERBA 200, Trans Asia, Mumbai, India. The intra- and inter-batch coefficients of variation (CVs) were < 5%. HbA1c was measured using high performance liquid chromatography (Bio-Rad D10; Bio-Rad Laboratories, Hercules, CA, USA) calibrated against the National Glycosylated Standardization Program with a CV of 2.8%. Fasting insulin was measured on an Abbott Architect i1000SR with a CV of 2.0%. Vitamin B_12_ (VitB12), folate, and vitamin D (VitD) were measured on the Abbott Architect i1000SR. The intra- and inter-batch CVs were 7.1% for VitB12, 7.7% for folate, and 5.3% for VitD. Total cholesterol, HDL, and triglycerides (TG) were measured on Trans Asia ERBA 200. The intra- and inter-batch CVs were 3.7% for CHOL, 5.2% for HDL, and 4.0% for TG.

### Calculations and classifications

Stunting and underweight (low Body Mass Index, BMI) were defined using World Health Organization (WHO) criteria (45) and thinness using International Obesity Task Force (IOTF) criteria (46), respectively. The Friedewald formula was used to calculate LDL (47). Prediabetes was defined using American Diabetic Association (ADA) criteria, i.e., fasting glucose ≥100 mg/dL or HbA1C ≥5.7% (48). Glycemic indices of insulin resistance (HOMa-IR), insulin sensitivity (HOMA-S) and beta cell function (HOMA-β) were estimated using the homeostasis model (49).

Elevated CHOL, LDL, and TG concentrations were defined as ≥ 200 mg/dL, ≥ 130 mg/dL, and ≥ 130 mg/dL, respectively, and low HDL was defined as < 40 mg/dL (28). Deficiency of vitB12 and folate was defined as <203 pg/mL and < 4.0 ng/mL (50), respectively. Deficiency of VitD was defined as <12 ng/mL (28).

### Statistical methods

Data has been represented by median and 25th–75th quartiles as well as by mean and standard deviation for continuous variables and by percentages for categorical variables. All the micronutrients (VitB12, folate, and VitD), lipids (CHOL, HDL, LDL, and TG), and all the glycemic variables (fasting glucose, fasting insulin, HOMA-IR, HOMA-S, and HOMA-β) were tested for normality. Except for fasting glucose, all the variables were skewed and appropriately transformed for normality. The transformation function to normalize was a natural logarithm for CHOL, HDL, TG, VitB12, HOMA-IR, and HOMA-S. The cube root function was used to normalize LDL and VitD. Folate and fasting insulin were normalized using hyperbolic arcsin functions. HOMA-β was normalized by subtracting the reciprocal of its square root from 1. Prediabetes, fasting insulin, and glycemic indices (HOMA-IR, HOMA-S, and HOMA-β) were treated as glycemic outcomes. Exposures refer to micronutrients (VitB12, VitD, and folate) and lipids (CHOL, LDL, HDL, and TG). Univariate associations between continuous glycemic outcomes and continuous exposures, as well as those between various continuous exposures (micronutrients and lipids), are shown by partial correlation. Comparison of exposures between normoglycemic and prediabetic girls was done by the analysis of variance for continuous and normally distributed exposures, by Mann–Whitney test for those not normally distributed and by chi-square test for those categorical. Prediabetes was a categorical outcome. Other categorical outcomes were defined using the presence of individuals in risk quartiles for each outcome. The risk quartiles for outcomes were the 1st quartile for fasting insulin and HOMA-β representing a group with poor insulin secretion and poor β cell function, respectively, and the 4th quartile for HOMA-IR and HOMA-S representing the most insulin resistant and most insulin-sensitive groups, respectively. We also categorized the exposures (micronutrients as well as lipids) by creating the quartiles of each exposure. The 4th quartile was used as a reference for VitB12, folate, and VitD, which represent high vitamin concentrations. Except for HDL, the 1st quartile was used as a reference for lipids (CHOL, LDL, and TG), representing low lipid levels. For HDL, the 4th quartile was used as a reference, indicating high or better HDL. Univariate as well as multivariate associations of the categorical outcomes with the categorical exposures were analyzed using logistic regression. Odds ratios (ORs) relative to the reference quartile for each exposure and 95% confidence intervals (CIs) for the outcomes were calculated. We also tested the interaction of various micronutrient and lipid exposures for various outcomes by including relevant product terms as appropriate. BMI representing the anthropometric markers of undernutrition and age of adolescent girls, was divided into quartiles and used as covariates. In the case of BMI, the 4th quartile was further divided into two groups: non-obese and overweight/obese, thus creating five groups. The overweight/obese group was treated as the reference. We also carried out the analysis for prediabetes using micronutrients and lipids as scale variables. Two-tailed significance was calculated at a 5% level. Analysis was performed using Statistical Package for the Social Sciences (SPSS) 25.0 and STATA 11.0 (STATA, College Station, TX, USA).

### Ethics

The study was approved by the Institute Ethics Committee of BKL Walawalkar Rural Medical College and Hospital. The committee is registered with the Department of Health Research (DHR), Government of India, with registration number EC/NEW/INST/2023/MH/0361. Appropriate written informed consent was obtained from those who were 18 years old at the time of the recruitment. For those below 18 years of age, written informed consent was obtained from the parents of the adolescent girl, and written informed ascent was obtained from the adolescent girl.

## Results

We recruited 1,520 adolescent girls in the cohort. Body composition measurements were done on 1,400 girls. Of this, five girls were diagnosed with diabetes. Out of the remaining normoglycemic (*n* = 1,395) cases, lipid and micronutrient measurements were available on 1,387 girls. Our final sample number for the data analysis is 1,387.

Anthropometry, body composition, micronutrients, lipids, and glycemia (Table 1).

**Table 1:** Anthropometry, body composition, micronutrients, lipids, and glycemia in adolescent girls (*n* = 1,387).

Parameters	Median (25th – 75th percentile) or n (%)	Mean (SD)
Body composition		
Age (yrs)	16.6 (15.8–17.3)	16.6 (0.9)
Height (cm)	151.7 (148.2–155.6)	151.8 (5.5)
Stunted^$^	**421 (30.4)**	
Weight (kg)	40.7 (36.7–46.0)	42.1 (8.1)
Underweight^$^	**400 (28.8)**	
BMI (kg/m^2^)	17.6 (16.0–19.8)	18.2 (3.3)
**Thinness** ^**@**^ **(< − 1 SD)**	**805 (58.0)**	
**Overweight/obese** ^**@**^ **(> + 1 SD)**	**60 (4.3)**	
Waist circumference (cm)	62.2 (58.5–67.2)	63.5 (7.3)
Hip circumference (cm)	83.2 (79.6–87.9)	84.2 (7.1)
WHR	0.75 (0.72–0.78)	0.75 (0.05)
Fat mass (kg)	8.9 (6.7–12.3)	10.2 (5.3)
Body fat %	22.5 (18.6–27.7)	23.4 (6.9)
**Body fat % > 25**	**504 (36.3)**	
Lean mass (kg)	29.5 (27.4–31.6)	29.6 (3.2)
**Vitamins**		
VitB12 (pg/mL)	249.0 (182.5–340.1)	287.3 (155.9)
**VitB12 < 203 pg/mL**	**445 (32.1)**	
Folate (ng/mL)	6.1 (4.7–7.8)	6.5 (2.6)
**Folate < 4 ng/mL**	**164 (11.8)**	
VitD (ng/mL)	14.2 (10.8–17.4)	14.6 (5.3)
**VitD ≤ 12 ng/mL**	**462 (33.3)**	
**Lipids**		
CHOL (mg/dL)	148.0 (131.0–165.0)	149.9 (27.3)
**CHOL ≥ 200 mg/dL**	**66 (4.8)**	
LDL (mg/dL)	81.5 (68.5–98.6)	84.5 (23.6)
**LDL ≥ 130 mg/dL**	**55 (4.0)**	
HDL (mg/dL)	50.8 (43.9–58.5)	51.7 (10.9)
**HDL < 40 mg/dL**	**177 (12.8)**	
TG (mg/dL)	61.5 (47.0–82.7)	68.5 (29.6)
**TG ≥ 130 mg/dL**	**53 (3.8)**	
**Glycemia**		
Fasting glucose (mg/dL)	95.4 (88.9–101.6)	95.1 (9.8)
**IFG**	**433 (31.2)**	
HbA_1_C (%)	5.3 (5.0–5.5)	5.2 (0.4)
**Elevated HbA**_**1**_ **C**	**191 (13.8)**	
**Prediabetes**	**551 (39.7)**	
Fasting insulin (μIU/mL)	8.6 (6.9–11.1)	9.3 (3.8)
HOMA-IR	1.2 (0.9–1.5)	1.2 (0.5)
HOMA-S	86.0 (66.5–108.5)	90.8 (36.3)
HOMA-β	94.5 (79.4–113.6)	100.4 (33.3)

^$^As per WHO (36); ^@^As per IOTF (International Obesity Task Force) (37); WHR, waist circumference to hip circumference ratio; SD, Standard Deviation; IFG, Impaired Fasting Glucose (fasting glucose ≥ 100 mg/dL); elevated HbA_1_C (>5.7%).

The median age of the subjects was 16.6 years. Median height, weight, BMI, waist circumference, hip circumference, and waist circumference to hip circumference ratio were 151.7 cm, 40.7 kg, 17.6 kg/m^2^, 62.2 cm, 83.2 cm, and 0.75, respectively. Using the WHO standard, 30.4% of girls were found to be stunted, and 28.8% were underweight. Using the IOTF standard, 58% were thin. Median body fat% was 22.5 and 36.3% girls had body fat% > 25. Median concentrations of VitB12, folate, and VitD were 249.0 pg/mL, 6.1 ng/mL, and 14.2 ng/mL, respectively. Deficiencies of VitB12, folate, and VitD were observed in 32.1, 11.8, and 33.3%, respectively. Median levels of CHOL, LDL, HDL, and TG were 148.0 mg/dL, 81.5 mg/dL, 50.8 mg/dL, and 61.5 mg/dL, respectively. Elevated levels were observed in 4.8% of CHOL, 4.0% of LDL, and 3.8% of TG. Low HDL was observed in 12.8%. Median concentrations of fasting glucose and fasting insulin were 95.4 mg/dL and 8.6 μIU/ml, respectively. Median values for glycemic indices were 1.2, 86.0, and 94.5 for HOMA-IR, HOMA-S, and HOMA-β, respectively. Prediabetes was observed in 39.7% of girls.

Micronutrients, lipids, and prediabetes (Table 2).

**Table 2:** Micronutrients and lipids between girls with PD and normoglycemia (*n* = 1,387).

Parameters	PD (*n* = 551)	Normal (*n* = 836)	*p* value
Micronutrients			
VitB12 (pg/mL)	256.0 (183.0–362.0)	244.9 (182.0–333.0)	0.118
<203 pg/mL	170 (30.9)	275 (32.9)	0.425
Folate (ng/mL)	6.0 (4.7–7.8)	6.1 (4.7–7.7)	0.877
<4 ng/mL	63 (11.4)	101 (12.1)	0.715
VitD (ng/mL)	14.0 (10.6–17.8)	14.2 (10.9–17.0)	0.952
≤12 ng/mL	189 (34.3)	273 (32.7)	0.525
Lipids			
CHOL (mg/dL)	150.0 (132.0–169.0)	146.8 (130.0–164.0)	0.013^*^
≥200 mg/dL	30 (5.4)	36 (4.3)	0.330
LDL (mg/dL)	85.1 (70.1–100.6)	79.8 (67.5–96.9)	0.004^*^
≥130 mg/dL	26 (4.7)	29 (3.5)	0.243
HDL (mg/dL)	51.2 (43.3–58.5)	50.6 (44.1–58.4)	0.855
<40 mg/dL	74 (13.4)	103 (12.3)	0.545
TG (mg/dL)	63.7 (47.7–84.3)	60.7 (46.3–82.3)	0.199
≥130 mg/dL	26 (4.7)	27 (3.2)	0.157

Median (25th – 75th percentile) or *n* (%); ^*^statistically significant (*p* < 0.05) by Man–Whitney Test for non-normal variables and chi-square test for categorical variables.

We compared micronutrient concentrations and lipids between prediabetic and normoglycemic girls. Among lipids, CHOL and LDL were higher in girls with PD (*p* < 0.01) for CHOL and (*p* < 0.001) for LDL.

Univariate and continuous associations of micronutrients and lipids with glycemic outcomes and BMI (Supplementary Table 1).

Folate was inversely associated with fasting insulin, HOMA-IR (*p* < 0.05 for both), and HOMA-β (*p* < 0.001). Total cholesterol and LDL were positively associated with fasting glucose (*p* < 0.05 for both). Total cholesterol, LDL, and TG were positively associated with fasting insulin and HOMA-IR and inversely with HOMA-S (*p* < 0.01 for all). HDL was inversely associated with fasting insulin and HOMA-IR and positively with HOMA-S (*p* < 0.001) for all. Triglyceride was positively associated with HOMA-β and HDL was inversely associated (*p* < 0.001 for both). All three micronutrients and HDL were inversely associated with BMI, and CHOL, LDL and TG were positively associated.

Univariate and continuous associations between micronutrients and lipids (Supplementary Table 2).

Vitamin B_12_ was positively associated with CHOL, LDL, and HDL but inversely with TG (*p* < 0.001 for all). Folate was inversely associated with LDL (*p* < 0.001) and positively with HDL (*p* < 0.05). Vitamin D was inversely associated with both CHOL and LDL (*p* < 0.01 for both).

Univariate analysis of micronutrients, lipids, BMI, and age as predictors of glycemic outcomes (Table 3).

**Table 3:** Association of PD and other glycemic outcomes with micronutrients, lipids, BMI, and age (univariate logistic regressions).

Exposures ↓	Prediabetes	Poor insulin secretion (Q1 of fasting insulin)	High HOMA-IR (Q4 of insulin resistance)	High HOMA-S (Q4 of insulin sensitivity)	Poor HOMA-β (Q1 of β cell function)
VitB12 Q1	NS	NS	NS	NS	NS
Q2	0.708 (0.522–0.960)0.026	NS	NS	NS	NS
Q3	NS	NS	NS	NS	NS
Q4 (ref)	1	1	1	1	1
Folate Q1	NS	NS	NS	NS	0.705 (0.499–0.997)0.048
Q2	NS	NS	NS	NS	0.710 (0.506–0.996)0.047
Q3	NS	NS	0.602 (0.410–0.884)0.010	NS	NS
Q4 (ref)	1	1	1	1	1
VitD Q1	NS	NS	NS	NS	NS
Q2	NS	NS	1.494 (1.050–2.127)0.026	NS	NS
Q3	0.687 (0.506–0.933)0.016	NS	NS	NS	NS
Q4 (ref)	1	1	1	1	1
CHOL Q4	1.513 (1.117–2.049)0.008	0.626 (0.438–0.895)0.010	1.661 (1.163–2.373)0.005	0.564 (0.396–0.805)0.002	NS
Q3	NS	NS	NS	NS	NS
Q2	NS	NS	NS	NS	NS
Q1 (ref)	1	1	1	1	1
LDL Q4	NS	NS	1.915 (1.329–2.735)0.000	NS	NS
Q3	1.379 (1.016–1.872)0.039	NS	1.554 (1.070–2.255)0.020	NS	NS
Q2	1.412 (1.041–1.916)0.027	NS	1.251 (0.853–1.835)0.251	NS	NS
Q1 (ref)	1	1	1	1	1
HDL Q1	NS	0.640 (0.452–0.906)0.012	1.902 (1.320–2.740)0.001	0.684 (0.484–0.966)0.031	0.668 (0.475–0.941)0.029
Q2	NS	NS	NS	NS	0.706 (0.502–0.993)0.045
Q3	NS	NS	NS	NS	NS
Q4 (ref)	1	1	1	1	1
TG Q4	NS	0.370 (0.254–0.537)0.000	1.934 (1.351–2.767)0.000	0.380 (0.263–0.549)0.000	0.612 (0.428–0.874)0.007
Q3	NS	0.645 (0.461–0.904)0.011	1.539 (1.066–2.220)0.021	0.580 (0.413–0.815)0.002	NS
Q2	NS	NS	NS	NS	NS
Q1 (ref)	1	1	1	1	1
BMI Q1	NS	36.393 (4.983–265.798)0.000	0.047 (0.025–0.091)0.000	36.840 (5.044–269.059)0.000	33.791 (4.626–246.852)0.001
Q2	NS	27.021 (3.696–197.566)0.001	0.078 (0.041–0.146)0.000	27.384 (3.746–200.208)0.001	24.224 (3.311–177.214)0.002
Q3	NS	15.719 (2.142–115.372)0.007	0.096 (0.052–0.179)0.000	16.268 (2.217–119.365)0.006	18.844 (2.572–138.091)0.004
Q4 – Normal	NS	NS	0.235 (0.128–0.433)0.000	NS	8.462 (1.137–62.975)0.037
Q4 – ovwt/obese (ref)	1	1	1	1	1
Age Q4	NS	1.686 (1.193–2.383)0.003	0.638 (0.458–946)0.024	1.709 (1.210–2.414)0.002	1.550 (1.108–2.169)0.010
Q3	NS	NS	NS	NS	NS
Q2	NS	NS	NS	NS	NS
Q1 (ref)	1	1	1	1	1

Values represent OR with 95% CI and an exact *p* value (<0.05); NS: Statistically not significant; Q1, Q2, Q3, and Q4 are quartiles of respective exposures; ovwt: Overweight; BMI Q4 is further divided into two groups: those with a normal BMI and those who are overweight/obese; ref: reference.

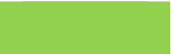
: represents the odds ratio < 1.

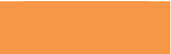
: represents the odds ratio > 1.

We carried out univariate logistic regressions using quartiles of each micronutrient and each lipid parameter, as well as 5 groups of increasing BMI and quartiles of age with PD and risk quartiles of fasting insulin and glycemic indices as outcomes.

Prediabetes: The OR for the 2nd quartile of VitB12 and the 3rd quartile of VitD were significantly reduced (<1) to the upper quartile, representing relatively high levels of respective vitamins. Among lipids, PD was predicted by high CHOL (4th quartile) and high LDL (2nd and 3rd quartiles) to the 1st quartile, representing low levels of respective lipids. There was no association of PD with HDL and TG.

Poor insulin secretion (1st quartile of fasting insulin): Having poor insulin secretion was associated inversely with high CHOL (4th quartile), low HDL (1st quartile), and high TG (3rd and 4th quartiles), with the 1st quartile representing low levels of CHOL, TG, and the 4th quartile representing high HDL. The OR for poor insulin secretion increased with decreasing BMI. Those who were the oldest (4th quartile) had a higher likelihood of poor insulin secretion than those who were the youngest.

Most insulin resistant (4th quartile of HOMA-IR): Among micronutrients, being most insulin resistant was inversely associated with folate (3rd quartile) and directly with VitD (2nd quartile). Among lipids, being most insulin resistant was associated with high CHOL (4th quartile), high LDL (2nd, 3rd, and 4th quartiles) low HDL (1st quartile), and high TG (3rd and 4th quartiles). The OR for being most insulin resistant decreased with decreasing BMI. Those who were oldest (4th quartile) had less likelihood of having most insulin resistance than those who were younger.

Most insulin sensitive (4th Quartile of HOMA-S): Protective associations were observed for high CHOL (4th quartile), low HDL (1st quartile), and high TG (3rd and 4th quartiles). The decreasing BMI increased the OR for being highly insulin sensitive. Those who were the oldest (4th quartile) had a high likelihood of being most insulin sensitive.

Poor β cell function (1st Quartile of HOMA-β): Poor β cell function was associated protectively with low folate (2nd and 1st quartile), low HDL (2nd and 1st quartile), and high TG (4th quartile). The OR for poor β cell function increased with decreasing BMI. Those who were the oldest (4th quartile) had a high likelihood of poor β cell function.

Multivariate analysis of micronutrients and lipids as predictors of glycemic outcomes.

We ran logistic regression models for each glycaemic outcome, containing a single micronutrient (VitB12/folate/VitD) and a single lipid (CHOL/LDL/HDL/TG) as independent variables and BMI and age as covariates. Thus, for each of the 3 micronutrients, 4 lipids, and 5 glycaemic outcomes, we ran 4 models for each of the 5 glycaemic outcomes. We also included relevant micronutrient and lipid interaction terms in the analysis.

Vitamin B_12_ and lipids as predictors of PD (Table 4).

**Table 4:** Multivariate association of PD with vitamin B_12_ and lipids with BMI and age as covariates (logistic regression).

Outcomes →	Prediabetes			
Exposures ↓	M_1_	M_2_	M_3_	M_4_
VitB12 Q1	NS	NS	NS	NS
Q2	NS	0.732 (0.538–0.996)0.047	0.708 (0.521–0.962)0.027	0.697 (0.514–0.947)0.021
Q3	NS	NS	NS	NS
Q4 (ref)	1	1	1	1
CHOL Q4	1.45 (1.06–1.98)0.020			
Q3	NS			
Q2	NS			
Q1 (ref)	1	1	1	1
LDL Q4		NS		
Q3		NS		
Q2		NS		
Q1 (ref)	1	1	1	1
HDL Q1			NS	
Q2			NS	
Q3			NS	
Q4 (ref)	1	1	1	1
TG Q4				NS
Q3				NS
Q2				NS
Q1 (ref)	1	1	1	1
BMI Q1	NS	NS	NS	NS
Q2	NS	NS	NS	NS
Q3	NS	NS	NS	NS
Q4 – Normal	NS	NS	NS	NS
Q4 – ovwt/obese (ref)	1	1	1	1
age Q4	NS	NS	NS	NS
Q3	NS	NS	NS	NS
Q2	NS	NS	NS	NS
Q1 (ref)	1	1	1	1

Values represent OR with 95% CI and an exact *p* value (<0.05); NS: Statistically not significant; Q1, Q2, Q3, and Q4 are quartiles of respective exposures; ovwt: Overweight; BMI Q4 is further divided into two groups: those with a normal BMI and those who are overweight/obese; ref: reference.

M_1_: Model-1 (VitB12, CHOL, BMI and age).

M_2_: Model-2 (VitB12, LDL, BMI and age).

M_3_: Model-3 (VitB12, HDL, BMI and age).

M_4_: Model-4 (VitaB12, TG, BMI and age).

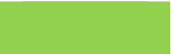
: represents the odds ratio < 1.

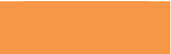
: represents the odds ratio > 1.

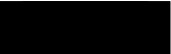
: variable not in the model.

In Model-M1 containing CHOL, PD was independently associated only with relatively high CHOL (4th quartile). In the remaining 3 models (M2, M3, and M4) containing each of the remaining lipids, PD was associated with only VitB12 (2nd quartile) and that too with protective effects (ORs < 1).

Folate and lipids as predictors of PD (Table 5).

**Table 5:** Multivariate association of PD with folate and lipids with BMI and age as covariates (logistic regression).

Outcomes →	Prediabetes			
Exposures ↓	M_1_	M_2_	M_3_	M_4_
Folate Q1	NS	NS	NS	NS
Q2	NS	NS	NS	NS
Q3	NS	NS	NS	NS
Q4 (ref)	1	1	1	1
CHOL Q4	1.507 (1.106–2.052)0.009			
Q3	NS			
Q2	NS			
Q1 (ref)	1			
LDL Q4		1.140 (1.026–1.911)0.034		
Q3		1.382 (1.015–1.881)0.040		
Q2		NS		
Q1 (ref)		1		
HDL Q1			NS	
Q2			NS	
Q3			NS	
Q4 (ref)			1	
TG Q4				NS
Q3				NS
Q2				NS
Q1 (ref)				1
BMI Q1	NS	NS	NS	NS
Q2	NS	NS	NS	NS
Q3	NS	NS	NS	NS
Q4 – Normal	NS	NS	NS	NS
Q4 – ovwt/obese (ref)	1	1	1	1
Age Q4	NS	NS	NS	NS
Q3	NS	NS	NS	NS
Q2	NS	NS	NS	NS
Q1 (ref)	1	1	1	1

Values represent OR with 95% CI and an exact *p* value (<0.05); NS: Statistically not significant; Q1, Q2, Q3, and Q4 are quartiles of respective exposures; ovwt: Overweight; BMI Q4 is further divided into two groups: those with a normal BMI and those who are overweight/obese; ref: reference.

M_1_: Model-1 (Folate, CHOL, BMI, and age).

M_2_: Model-2 (Folate, LDL, BMI, and age).

M_3_: Model-3 (Folate, HDL, BMI, and age).

M_4_: Model-4 (Folate, TG, BMI, and age).

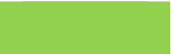
: represents the odds ratio < 1.

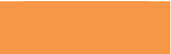
: represents the odds ratio > 1.

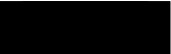
: variable not in the model.

In Model-1 containing CHOL, PD was independently associated only with relatively high cholesterol (4th quartile). In the remaining models, there was an independent association of PD only with relatively high LDL (3rd and 4th quartiles) in Model-2.

Vitamin D and lipids as predictors of PD (Table 6).

**Table 6:** Multivariate association of PD with vitamin D and lipids with BMI and age as covariates (logistic regression).

Outcomes →	Prediabetes			
Exposures ↓	M_1_	M_2_	M_3_	M_4_
VitD Q1	NS	NS	NS	NS
Q2	NS	NS	NS	NS
Q3	0.669 (0.491–0.911)0.011	0.667 (0.493–0.909)0.010	0.671 (0.493–0.913)0.011	0.664 (0.487–0.904)0.009
Q4 (ref)	1	1	1	1
CHOL Q4	1.523 (1.117–2.017)0.008			
Q3	NS			
Q2	NS			
Q1 (ref)	1			
LDL Q4		1.405 (1.028–1.920)0.033		
Q3		1.403 (1.029–1.910)0.032		
Q2		NS		
Q1 (ref)		1		
HDL Q1			NS	
Q2			NS	
Q3			NS	
Q4 (ref)			1	
TG Q4				NS
Q3				NS
Q2				NS
Q1 (ref)				1
BMI Q1	NS	NS	NS	NS
Q2	NS	NS	NS	NS
Q3	NS	NS	NS	NS
Q4: Normal	NS	NS	NS	NS
Q4: ovwt/obese (ref)	1	1	1	1
age Q4	NS	NS	NS	NS
Q3	NS	NS	NS	NS
Q2	NS	NS	NS	NS
Q1 (ref)	1	1	1	1

Values represent OR with 95% CI and an exact *p* value (<0.05); NS: Statistically not significant; Q1, Q2, Q3, and Q4 are quartiles of respective exposures; ovwt: Overweight; BMI Q4 is further divided into two groups: those with a normal BMI and those who are overweight/obese; ref: reference.

M_1_: Model-1 (VitD, CHOL, BMI, and age).

M_2_: Model-2 (VitD, LDL, BMI, and age).

M_3_: Model-3 (VitD, HDL, BMI, and age).

M_4_: Model-4 (VitD, TG, BMI, and age).

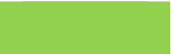
: represents the odds ratio < 1.

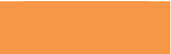
: represents the odds ratio > 1.

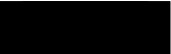
: variable not in the model.

In all 4 models, PD was independently associated with VitD (3rd quartile) and protective OR. PD was also independently associated with high cholesterol (4th quartile) in Model-1 and with high LDL (3rd and 4th quartiles) in Model-2.

We repeated the analysis described in Tables 4–6 for the remaining glycemic outcomes, i.e., poor insulin secretion, being most insulin resistant, being most insulin sensitive, and having poor β cell function. We have summarized these in Supplementary Tables 3–5 containing vitamin B_12_, folate, and vitamin D, respectively.

Vitamin B_12_ and lipids as predictors of poor insulin secretion (Supplementary Table 3).

Having poor insulin secretion was independently associated with poor VitB12 status (1st quartile) in all 4 models. The ORs were significant and >1 in all. Additionally, in Model-4 containing TG, there was also an independent association with elevated TG (4th quartile), but the effect was protective as OR was significant and <1.

Folate and lipids as predictors of poor insulin secretion (Supplementary Table 4).

Only in Model-4 containing TG was there an independent association with elevated TG (4th quartile), but the effect was protective as OR was significant and <1.

Vitamin D and lipids as predictors of poor insulin secretion (Supplementary Table 5).

Only in Model-4 containing TG was there an independent association with elevated TG (4th quartile), but the effect was protective as OR was significant and <1.

Vitamin B_12_ and lipids as predictors of being most insulin resistant (Supplementary Table 3).

The only association observed was in Model-3 with low HDL (1st quartile), and the OR was significant and >1.

Folate and lipids as predictors of being most insulin resistant (Supplementary Table 4).

There was a protective effect of low folate (3rd quartile) in all 4 models, as ORs were significant and <1. Additionally, there was a positive effect of low HDL (1st quartile) in Model-3.

Vitamin D and lipids as predictors of being most insulin resistant (Supplementary Table 5).

The only significant positive effect observed was that of low HDL (1st quartile) in Model-3.

Vitamin B_12_ and lipids as predictors of being most insulin sensitive (Supplementary Table 3).

Only in Model-4, a significant but protective effect of high TG (4th quartile) was observed.

Folate and lipids as predictors of being most insulin sensitive (Supplementary Table 4).

The protective effect of high CHOL (4th quartile) in Model-1 was observed. A significant but protective effect of high TG (4th quartile) was also observed in Model-4.

Vitamin D and lipids as predictors of being most insulin sensitive (Supplementary Table 5).

The protective effect of high CHOL (4th quartile) and high TG (4th quartile) was observed in Model-1 and Model-4, respectively.

Vitamin B_12_ and lipids as predictors of poor β cell function (Supplementary Table 3).

There was a positive effect of high LDL (4th quartile) in Model-2 and a protective effect of low HDL (2nd quartile) in Model-3.

Folate and lipids as predictors of poor β cell function (Supplementary Table 4).

Folate (2nd quartile) had a protective effect on poor β cell function in all models. There was a positive effect of high LDL (4th quartile) in Model-2 and a protective effect of low HDL (2nd quartile) in Model-3.

Vitamin D and lipids as predictors of poor β cell in Model-3 function (Supplementary Table 5).

The likelihood of poor β cell function was not associated with any of the lipids.

Interactions of micronutrients and lipids with prediabetes, poor insulin secretion, and glycemic indices.

None of the interactions between each micronutrient and each lipid were found to be statistically significant for PD and other glycemic outcomes.

We also carried out multivariate logistic regression of PD, including independent variables such as lipids and micronutrients as scale variables (Supplementary Tables 6–8) without using quartiles. The results were very similar. Prediabetes was associated with CHOL and LDL.

## Discussion

We have reported micronutrients (VitB12, folate, and VitD) and lipid levels (CHOL, LDL, HDL, and TG) in 16–18 year-old adolescent girls of the DERVAN cohort. Among micronutrients, more than 1/3rd of girls were deficient in VitB12 and VitD. This was very similar to those reported in a national survey of adolescents (28). Folate deficiency was very low. Elevated CHOL, LDL, and TG were observed in <5%, but low HDL was observed in 12.8%. This was despite household chores and walking long distances to school (51). The prevalence of PD was close to 40%. Abnormality in lipids using the conventional cutoffs was itself very low. Prediabetes was not associated with micronutrient deficiencies when we used conventional cutoffs. Unlike other reports from India (21), the proportion of lipid abnormalities between prediabetics and normal was similar and very low. Cutoffs for micronutrient deficiencies are also facing challenges in Indian settings (52). Hence, we decided to use a quartile approach for both sets of exposures to test the associations of glycemic outcomes with micronutrients as well as lipid exposures. Other than PD, there are no specific cutoffs for insulin and various glycemic indices; hence, we also categorized all the outcomes other than PD as belonging to either the lowest or highest quartiles.

In the multivariate analysis using a quartile approach, relatively low VitB12 and VitD status were protective against PD. Among lipids, the likelihood of PD was high in those with relatively high CHOL, and it was much stronger in those with relatively high LDL. The likelihood of poor insulin secretion increased in those with relatively low VitB12, while it decreased in those with relatively high TG. Relatively low folate status was protective against high insulin resistance, while a high likelihood of insulin resistance was observed in those with relatively low HDL. Those with relatively high TG were less likely to have high insulin sensitivity, and those with relatively high LDL and low HDL were more likely to have poor β cell function. It is noteworthy that despite the very low prevalence of those with abnormal CHOL and LDL, there was still a graded association of PD with those having relatively high CHOL and LDL, independent of all three micronutrients.

Interactions between micronutrients and lipids have been reported for PD in another report from India on school-going young children of 5–9 years of age (53). It found significant interaction probabilities for PD among those with VitB12 deficiency with high CHOL, Sufficient VitD with high CHOL, and high VitD with high LDL. We used the odds ratio approach and found high likelihood of PD among those with relatively high CHOL, and relatively low VitD with high CHOL. The interesting thing in our data was the absence of statistical interactions between micronutrients and lipids for various glycemic outcomes. Beyond PD, the measurement of insulin and glycemic indices provided the opportunity to explore their associations with micronutrients and lipids. We could not find any reports that investigated such associations and interactions.

There are few studies reporting data on micronutrients, lipids, prediabetes, and diabetes in adolescent populations. A study from Italy (54) found an association between poor vitamin B_12_ and high insulin resistance. A study from Saudi Arabia (55) found a significant association of vitamin D deficiency with T2D. The prevalence of overweight/obesity in these studies was 34 and 68%, respectively. The proportion of those overweight/obese in our study was miniscule (only 4.3%). Both studies have measured lipids, but none of them have reported the associations of diabetes or prediabetes with micronutrients and lipids together. Unlike our study, these studies have measured only a single micronutrient.

The prevalence of various lipid abnormalities that contribute to dyslipidemia was very low in our cohort. Lipids in non-pregnant adult populations always receive much attention due to their known associations with CVD as well as NCDs, but they are also known to play an important role as fuels in pregnancy (56). Our cohort consists of adolescent girls. Lipid levels in adolescence are known to track with those in later life (22, 23). Thus, our girls are likely to begin the pregnancy with inadequate levels of fuel/lipids. In 1980, Freinkel introduced the concept of ‘Fuel-mediated teratogenesis,’ (56) where the mixture of maternal nutrients/fuels (glucose and lipids) affects not only fetal growth but also the risk of future obesity and diabetes. Maternal lipids (CHOL and TG) are essential for fetal development (57, 58) and their low levels during pregnancy have been associated with delayed prenatal growth (59, 60). Low birthweight and stunting continue to be high in our region (61, 62). Studies from the US (63) and Europe (64–66) have shown associations between maternal lipids in pregnancy and birth weight. A study among undernourished rural pregnant Indian women has also shown a strong association between maternal glucose and lipids and fetal growth (67). The high prevalence of PD in our cohort (43) has already put our girls at risk of developing gestational diabetes. Thus, our adolescent girls are likely to enter pregnancy with risks of hyperglycemia, micronutrient deficiencies, and poor fuel storage. Prediabetes in our girls is driven by poor insulin secretion and poor β cell function. Based on our findings, improving vitB12, maintaining adequate TG, and reducing LDL in adolescence may help improve insulin secretion as well as β-cell function, leading to a reduction in PD. This indicates the need for lipid and micronutrient-based interventions in adolescents to improve glycemic outcomes. Maintaining adequate storage of micronutrients as well as fuels preconceptionally could reduce NCD risks. However, caution is warranted, as excess lipids may contribute to future obesity and diabetes-related insulin resistance.

The strengths of our study are the large sample size and measurements of detailed glycemic parameters beyond glucose. We measured micronutrients as well as lipids in a region known for widespread undernutrition across the life course. The prevalence of overweight/obesity in our sample was extremely low. There are some limitations, too. Our cohort consists only of girls. We did not measure other vitamins, like B_2_ and B_6_.

There are many lipid-based nutrient supplementation (LNS) studies in young malnourished children (68–70), but LNS studies among undernourished women with a life-course approach spanning from adolescence to pregnancy and adulthood are needed to assess their impact on diabetes risks in individuals as well as in the next generation.

To summarize, our report on adolescent girls has attempted to shed some light on the possible role of not only micronutrients but also lipids in undernourished adolescent girls on the development of NCDs in adulthood as well as in the next generation.

## Conclusion

In short, we have shown the existence of micronutrient deficiencies and poor lipid stores among rural Indian adolescent girls. We have also demonstrated their link to prediabetes. A link between micronutrient undernutrition in early life and diabetes in adulthood is well studied. Lipids play an important role as fuel in pregnancy. The persistence of poor lipid stores in adolescent girls, together with prediabetes, is likely to result in poor birth outcomes, like low birth weight. Lipid-based supplementation in adolescence together, with micronutrients, may offer a window of opportunity to reduce the subsequent risks of poor birth outcomes and diabetes in later life.

## Data availability statement

The datasets presented in this article are not readily available because the datasets generated are available from the corresponding author on reasonable request with necessary permissions from Institutional Ethics Committee and Government of India HMSC. Requests to access the datasets should be directed to SP, dr.suvrnanpatil@gmail.com. Institutional Ethics Committee and Government.

## Ethics statement

The studies involving humans were approved by Institute Ethics Committee of BKL Walawalkar Rural Medical College and Hospital. Registration number is (EC/NEW/INST/2023/MH/0361). The studies were conducted in accordance with the local legislation and institutional requirements. Written informed consent for participation in this study was provided by the participants’ legal guardians/next of kin.

## Author contributions

SP: Conceptualization, Funding acquisition, Supervision, Writing – original draft, Project administration, Writing – review & editing. OD: Data curation, Formal analysis, Software, Writing – original draft. PH-B: Conceptualization, Investigation, Methodology, Validation, Visualization, Writing – original draft. CJ: Conceptualization, Formal analysis, Methodology, Visualization, Writing – original draft, Writing – review & editing. RB: Validation, Writing – review & editing. NP: Funding acquisition, Resources, Writing – review & editing. AY: Project administration, Investigation, Methodology, Writing – review & editing.
