# Supplementary material for: Associations of micronutrients and lipids with prediabetes and glycemic parameters in adolescent girls of the rural DERVAN cohort (DERVAN-9)
Source: Front Nutr. 2024 Jul 11;11:1380777. doi: 10.3389/fnut.2024.1380777 (PMC11271042; doi:10.3389/fnut.2024.1380777)
Supplement: Supplementary file 1 [file Table_1.DOCX]

**Supplementary table 1:** Partial correlations of micronutrients and lipids with glycemic parameters and BMI. (n=1387)

|  | **Fasting glucose**  **(mg/dl)** | **Fasting**  **Insulin**  **(µIU/ml)** | **HOMA-IR** | **HOMA-S** | **HOMA-β** | **BMI**  **(kg/m^2^)** |
| --- | --- | --- | --- | --- | --- | --- |
| **Vitamins** |  |  |  |  |  |  |
| ViB12 (pg/ml) | 0.00 | 0.00 | 0.00 | 0.00 | 0.00 | -0.14^***^ |
| Folate (ng/ml) | 0.04 | -0.06^*^ | -0.06^*^ | 0.05 | -0.07^**^ | -0.13^***^ |
| VitD (ng/ml) | -0.02 | -0.04 | -0.05 | 0.04 | -0.02 | -0.07^**^ |
| **Lipids** |  |  |  |  |  |  |
| CHOL (md/dl) | 0.05^*^ | 0.09^**^ | 0.09^**^ | -0.09^**^ | 0.04 | 0.14^***^ |
| LDL (mg/dl) | 0.06^*^ | 0.09^**^ | 0.10^***^ | -0.10^***^ | 0.04 | 0.15^***^ |
| HDL (mg/dl) | 0.01 | -0.10^***^ | -0.10^***^ | 0.10^***^ | -0.09^***^ | -0.11^***^ |
| TG (mg/dl) | 0.01 | 0.19^***^ | 0.20^***^ | -0.19^***^ | 0.16^***^ | 0.25^***^ |

^*^p<0.05; ^**^p<0.01; ^***^p<0.001; adjusted for age

**Supplementary table 2:** Partial correlations between lipids and micronutrients. (n=1387)

|  | **VitB12 (pg/ml)** | **Folate (ng/ml)** | **VitD (ng/ml)** |
| --- | --- | --- | --- |
| CHOL (md/dl) | 0.13^***^ | -0.04 | -0.06^*^ |
| LDL (mg/dl) | 0.14^***^ | -0.09^**^ | -0.08^**^ |
| HDL (mg/dl) | 0.09^***^ | 0.06^*^ | -0.01 |
| TG (mg/dl) | -0.12^***^ | 0.04 | 0.01 |

^*^p<0.05; ^**^p<0.01; ^***^p<0.001; adjusted for age

**Supplementary table 3:** Vitamin B_12_, lipids and glycemic outcomes.

| **Outcomes →** | **Prediabetes** | | | | **Poor insulin secretion** | | | | **High HOMA-IR** | | | | **High HOMA-S** | | | | **Poor HOMA-β** | | | | |
| --- | --- | --- | --- | --- | --- | --- | --- | --- | --- | --- | --- | --- | --- | --- | --- | --- | --- | --- | --- | --- | --- |
| **Exposures ↓** | **M_1_** | **M_2_** | **M_3_** | **M_4_** | **M_1_** | **M_2_** | **M_3_** | **M_4_** | **M_1_** | **M_2_** | **M_3_** | **M_4_** | **M_1_** | **M_2_** | **M_3_** | **M_4_** | **M_1_** | **M_2_** | **M_3_** | **M_4_** |  |
| VitB12 Q1 |  |  |  |  |  |  |  |  |  |  |  |  |  |  |  |  |  |  |  |  |  |
| VitB12 Q2 |  |  |  |  |  |  |  |  |  |  |  |  |  |  |  |  |  |  |  |  |  |
| VitB12 Q3 |  |  |  |  |  |  |  |  |  |  |  |  |  |  |  |  |  |  |  |  |  |
| VitB12 Q4 (ref) | 1 | 1 | 1 | 1 | 1 | 1 | 1 | 1 | 1 | 1 | 1 | 1 | 1 | 1 | 1 | 1 | 1 | 1 | 1 | 1 |  |
| CHOL Q4 |  |  |  |  |  |  |  |  |  |  |  |  |  |  |  |  |  |  |  |  |  |
| CHOL Q3 |  |  |  |  |  |  |  |  |  |  |  |  |  |  |  |  |  |  |  |  |  |
| CHOL Q2 |  |  |  |  |  |  |  |  |  |  |  |  |  |  |  |  |  |  |  |  |  |
| CHOL Q1 (ref) | 1 |  |  |  | 1 |  |  |  | 1 | 1 | 1 | 1 | 1 | 1 | 1 | 1 | 1 | 1 | 1 | 1 |  |
| LDL Q4 |  |  |  |  |  |  |  |  |  |  |  |  |  |  |  |  |  |  |  |  |  |
| LDL Q3 |  |  |  |  |  |  |  |  |  |  |  |  |  |  |  |  |  |  |  |  |  |
| LDL Q2 |  |  |  |  |  |  |  |  |  |  |  |  |  |  |  |  |  |  |  |  |  |
| LDL Q1 (ref) | 1 | 1 | 1 | 1 | 1 | 1 | 1 | 1 | 1 | 1 | 1 | 1 | 1 | 1 | 1 | 1 | 1 | 1 | 1 | 1 |  |
| HDL Q1 |  |  |  |  |  |  |  |  |  |  |  |  |  |  |  |  |  |  |  |  |  |
| HDL Q2 |  |  |  |  |  |  |  |  |  |  |  |  |  |  |  |  |  |  |  |  |  |
| HDL Q3 |  |  |  |  |  |  |  |  |  |  |  |  |  |  |  |  |  |  |  |  |  |
| HDL Q4 (ref) | 1 | 1 | 1 | 1 | 1 | 1 | 1 | 1 | 1 | 1 | 1 | 1 | 1 | 1 | 1 | 1 | 1 | 1 | 1 | 1 |  |
| TG Q4 |  |  |  |  |  |  |  |  |  |  |  |  |  |  |  |  |  |  |  |  |  |
| TG Q3 |  |  |  |  |  |  |  |  |  |  |  |  |  |  |  |  |  |  |  |  |  |
| TG Q2 |  |  |  |  |  |  |  |  |  |  |  |  |  |  |  |  |  |  |  |  |  |
| TG Q1 (ref) | 1 | 1 | 1 | 1 | 1 | 1 | 1 | 1 | 1 | 1 | 1 | 1 | 1 | 1 | 1 | 1 | 1 | 1 | 1 | 1 |  |
| BMI Q1 |  |  |  |  |  |  |  |  |  |  |  |  |  |  |  |  |  |  |  |  |  |
| BMI Q2 |  |  |  |  |  |  |  |  |  |  |  |  |  |  |  |  |  |  |  |  |  |
| BMI Q3 |  |  |  |  |  |  |  |  |  |  |  |  |  |  |  |  |  |  |  |  |  |
| BMI Q4 - Normal |  |  |  |  |  |  |  |  |  |  |  |  |  |  |  |  |  |  |  |  |  |
| BMI Q4 - ovwt/obese (ref) | 1 | 1 | 1 | 1 | 1 | 1 | 1 | 1 | 1 | 1 | 1 | 1 | 1 | 1 | 1 | 1 | 1 | 1 | 1 | 1 |  |
| age Q4 |  |  |  |  |  |  |  |  |  |  |  |  |  |  |  |  |  |  |  |  |  |
| age Q3 |  |  |  |  |  |  |  |  |  |  |  |  |  |  |  |  |  |  |  |  |  |
| age Q2 |  |  |  |  |  |  |  |  |  |  |  |  |  |  |  |  |  |  |  |  |  |
| age Q1 (ref) | 1 | 1 | 1 | 1 | 1 | 1 | 1 | 1 | 1 | 1 | 1 | 1 | 1 | 1 | 1 | 1 | 1 | 1 | 1 | 1 |  |

Values represents odds ratios with 95% confidence intervals and exact p value (< 0.05); NS: Statistically not significant; Q1, Q2, Q3, Q4 are quartiles of respective exposures; ovwt: Overweight; BMI Q4 is further divided into two groups of normal BMI and those overweight/obese; ref: reference.

M_1_ : Model-1 (VitB12, CHOL, BMI and age); M_2_ : Model-2 (VitB12, LDL, BMI and age); M_3_ : Model-3 (VitB12, HDL, BMI and age);M_4_ : Model-4 (VitaB12, TG, BMI and age).

|  | represents odds ratio < 1; |
| --- | --- |
|  | represents odds ratio > 1; |
|  | Variable not in the model. |

**Supplementary table 4:** Folate, lipids and glycemic outcomes.

| **Outcomes →** | **Prediabetes** | | | | **Poor insulin secretion** | | | | **High HOMA-IR** | | | | | **High HOMA-S** | | | | | **Poor HOMA-β** | | | | |
| --- | --- | --- | --- | --- | --- | --- | --- | --- | --- | --- | --- | --- | --- | --- | --- | --- | --- | --- | --- | --- | --- | --- | --- |
| **Exposures ↓** | M_1_ | M_2_ | M_3_ | M_4_ | M_1_ | M_2_ | M_3_ | M_4_ | M_1_ | M_2_ | M_3_ | M_4_ | M_1_ | | M_2_ | M_3_ | M_4_ | M_1_ | | M_2_ | M_3_ | M_4_ |  |
| Folate Q1 |  |  |  |  |  |  |  |  |  |  |  |  |  | |  |  |  |  | |  |  |  |  |
| Folate Q2 |  |  |  |  |  |  |  |  |  |  |  |  |  | |  |  |  |  | |  |  |  |  |
| Folate Q3 |  |  |  |  |  |  |  |  |  |  |  |  |  | |  |  |  |  | |  |  |  |  |
| Folate Q4 (ref) | 1 | 1 | 1 | 1 | 1 | 1 | 1 | 1 | 1 | 1 | 1 | 1 | 1 | | 1 | 1 | 1 | 1 | | 1 | 1 | 1 |  |
| CHOL Q4 |  |  |  |  |  |  |  |  |  |  |  |  |  | |  |  |  |  | |  |  |  |  |
| CHOL Q3 |  |  |  |  |  |  |  |  |  |  |  |  |  | |  |  |  |  | |  |  |  |  |
| CHOL Q2 |  |  |  |  |  |  |  |  |  |  |  |  |  | |  |  |  |  | |  |  |  |  |
| CHOL Q1 (ref) | 1 |  |  |  | 1 |  |  |  | 1 | 1 | 1 | 1 | 1 | | 1 | 1 | 1 | 1 | | 1 | 1 | 1 |  |
| LDL Q4 |  |  |  |  |  |  |  |  |  |  |  |  |  | |  |  |  |  | |  |  |  |  |
| LDL Q3 |  |  |  |  |  |  |  |  |  |  |  |  |  | |  |  |  |  | |  |  |  |  |
| LDL Q2 |  |  |  |  |  |  |  |  |  |  |  |  |  | |  |  |  |  | |  |  |  |  |
| LDL Q1 (ref) | 1 | 1 | 1 | 1 | 1 | 1 | 1 | 1 | 1 | 1 | 1 | 1 | 1 | | 1 | 1 | 1 | 1 | | 1 | 1 | 1 |  |
| HDL Q1 |  |  |  |  |  |  |  |  |  |  |  |  |  | |  |  |  |  | |  |  |  |  |
| HDL Q2 |  |  |  |  |  |  |  |  |  |  |  |  |  | |  |  |  |  | |  |  |  |  |
| HDL Q3 |  |  |  |  |  |  |  |  |  |  |  |  |  | |  |  |  |  | |  |  |  |  |
| HDL Q4 (ref) | 1 | 1 | 1 | 1 | 1 | 1 | 1 | 1 | 1 | 1 | 1 | 1 | 1 | | 1 | 1 | 1 | 1 | | 1 | 1 | 1 |  |
| TG Q4 |  |  |  |  |  |  |  |  |  |  |  |  |  | |  |  |  |  | |  |  |  |  |
| TG Q3 |  |  |  |  |  |  |  |  |  |  |  |  |  | |  |  |  |  | |  |  |  |  |
| TG Q2 |  |  |  |  |  |  |  |  |  |  |  |  |  | |  |  |  |  | |  |  |  |  |
| TG Q1 (ref) | 1 | 1 | 1 | 1 | 1 | 1 | 1 | 1 | 1 | 1 | 1 | 1 | 1 | | 1 | 1 | 1 | 1 | | 1 | 1 | 1 |  |
| BMI Q1 |  |  |  |  |  |  |  |  |  |  |  |  |  | |  |  |  |  | |  |  |  |  |
| BMI Q2 |  |  |  |  |  |  |  |  |  |  |  |  |  | |  |  |  |  | |  |  |  |  |
| BMI Q3 |  |  |  |  |  |  |  |  |  |  |  |  |  | |  |  |  |  | |  |  |  |  |
| BMI Q4 - Normal |  |  |  |  |  |  |  |  |  |  |  |  |  | |  |  |  |  | |  |  |  |  |
| BMI Q4 - ovwt/obese (ref) | 1 | 1 | 1 | 1 | 1 | 1 | 1 | 1 | 1 | 1 | 1 | 1 | 1 | | 1 | 1 | 1 | 1 | | 1 | 1 | 1 |  |
| age Q4 |  |  |  |  |  |  |  |  |  |  |  |  |  | |  |  |  |  | |  |  |  |  |
| age Q3 |  |  |  |  |  |  |  |  |  |  |  |  |  | |  |  |  |  | |  |  |  |  |
| age Q2 |  |  |  |  |  |  |  |  |  |  |  |  |  | |  |  |  |  | |  |  |  |  |
| age Q4 (ref) | 1 | 1 | 1 | 1 | 1 | 1 | 1 | 1 | 1 | 1 | 1 | 1 | 1 | | 1 | 1 | 1 | 1 | | 1 | 1 | 1 |  |

Values represents odds ratios with 95% confidence intervals and exact p value (< 0.05); NS: Statistically not significant; Q1, Q2, Q3, Q4 are quartiles of respective exposures; ovwt: Overweight; BMI Q4 is further divided into two groups of normal BMI and those overweight/obese; ref: reference.

M_1_ : Model-1 (Folate, CHOL and BMI); M_2_ : Model-2 (Folate, LDL and BMI); M_3_ : Model-3 (Folate, HDL and BMI); M_4_ : Model-4 (Folate, TG and BMI).

|  | represents odds ratio < 1; |
| --- | --- |
|  | represents odds ratio > 1; |
|  | Variable not in the model. |

**Supplementary table 5:** Vitamin D, lipids and glycemic outcomes.

| **Outcomes →** | **Prediabetes** | | | | **Poor insulin secretion** | | | | **High HOMA-IR** | | | | **High HOMA-S** | | | | **Poor HOMA-β** | | | | |
| --- | --- | --- | --- | --- | --- | --- | --- | --- | --- | --- | --- | --- | --- | --- | --- | --- | --- | --- | --- | --- | --- |
| **Exposures ↓** | **M_1_** | **M_2_** | **M_3_** | **M_4_** | **M_1_** | **M_2_** | **M_3_** | **M_4_** | **M_1_** | **M_2_** | **M_3_** | **M_4_** | **M_1_** | **M_2_** | **M_3_** | **M_4_** | **M_1_** | **M_2_** | **M_3_** | **M_4_** |  |
| VitD Q1 |  |  |  |  |  |  |  |  |  |  |  |  |  |  |  |  |  |  |  |  |  |
| VitD Q2 |  |  |  |  |  |  |  |  |  |  |  |  |  |  |  |  |  |  |  |  |  |
| VitD Q3 |  |  |  |  |  |  |  |  |  |  |  |  |  |  |  |  |  |  |  |  |  |
| VitD Q4 (ref) | 1 | 1 | 1 | 1 | 1 | 1 | 1 | 1 | 1 | 1 | 1 | 1 | 1 | 1 | 1 | 1 | 1 | 1 | 1 | 1 |  |
| CHOL Q4 |  |  |  |  |  |  |  |  |  |  |  |  |  |  |  |  |  |  |  |  |  |
| CHOL Q3 |  |  |  |  |  |  |  |  |  |  |  |  |  |  |  |  |  |  |  |  |  |
| CHOL Q2 |  |  |  |  |  |  |  |  |  |  |  |  |  |  |  |  |  |  |  |  |  |
| CHOL Q1 (ref) | 1 |  |  |  | 1 |  |  |  | 1 | 1 | 1 | 1 | 1 | 1 | 1 | 1 | 1 | 1 | 1 | 1 |  |
| LDL Q4 |  |  |  |  |  |  |  |  |  |  |  |  |  |  |  |  |  |  |  |  |  |
| LDL Q3 |  |  |  |  |  |  |  |  |  |  |  |  |  |  |  |  |  |  |  |  |  |
| LDL Q2 |  |  |  |  |  |  |  |  |  |  |  |  |  |  |  |  |  |  |  |  |  |
| LDL Q1 (ref) | 1 | 1 | 1 | 1 | 1 | 1 | 1 | 1 | 1 | 1 | 1 | 1 | 1 | 1 | 1 | 1 | 1 | 1 | 1 | 1 |  |
| HDL Q1 |  |  |  |  |  |  |  |  |  |  |  |  |  |  |  |  |  |  |  |  |  |
| HDL Q2 |  |  |  |  |  |  |  |  |  |  |  |  |  |  |  |  |  |  |  |  |  |
| HDL Q3 |  |  |  |  |  |  |  |  |  |  |  |  |  |  |  |  |  |  |  |  |  |
| HDL Q4 (ref) | 1 | 1 | 1 | 1 | 1 | 1 | 1 | 1 | 1 | 1 | 1 | 1 | 1 | 1 | 1 | 1 | 1 | 1 | 1 | 1 |  |
| TG Q4 |  |  |  |  |  |  |  |  |  |  |  |  |  |  |  |  |  |  |  |  |  |
| TG Q3 |  |  |  |  |  |  |  |  |  |  |  |  |  |  |  |  |  |  |  |  |  |
| TG Q2 |  |  |  |  |  |  |  |  |  |  |  |  |  |  |  |  |  |  |  |  |  |
| TG Q1 (ref) | 1 | 1 | 1 | 1 | 1 | 1 | 1 | 1 | 1 | 1 | 1 | 1 | 1 | 1 | 1 | 1 | 1 | 1 | 1 | 1 |  |
| BMI Q1 |  |  |  |  |  |  |  |  |  |  |  |  |  |  |  |  |  |  |  |  |  |
| BMI Q2 |  |  |  |  |  |  |  |  |  |  |  |  |  |  |  |  |  |  |  |  |  |
| BMI Q3 |  |  |  |  |  |  |  |  |  |  |  |  |  |  |  |  |  |  |  |  |  |
| BMI Q4 - Normal |  |  |  |  |  |  |  |  |  |  |  |  |  |  |  |  |  |  |  |  |  |
| BMI Q4 - ovwt/obese (ref) | 1 | 1 | 1 | 1 | 1 | 1 | 1 | 1 | 1 | 1 | 1 | 1 | 1 | 1 | 1 | 1 | 1 | 1 | 1 | 1 |  |
| age Q4 |  |  |  |  |  |  |  |  |  |  |  |  |  |  |  |  |  |  |  |  |  |
| age Q3 |  |  |  |  |  |  |  |  |  |  |  |  |  |  |  |  |  |  |  |  |  |
| age Q2 |  |  |  |  |  |  |  |  |  |  |  |  |  |  |  |  |  |  |  |  |  |
| age Q4 (ref) | 1 | 1 | 1 | 1 | 1 | 1 | 1 | 1 | 1 | 1 | 1 | 1 | 1 | 1 | 1 | 1 | 1 | 1 | 1 | 1 |  |

Values represents odds ratios with 95% confidence intervals and exact p value (< 0.05); NS: Statistically not significant; Q1, Q2, Q3, Q4 are quartiles of respective exposures; ovwt: Overweight; BMI Q4 is further divided into two groups of normal BMI and those overweight/obese; ref: reference.

M_1_ : Model -1 (VitD, CHOL and BMI); M_2_ : Model -2 (VitD, LDL and BMI); M_3_ : Model -3 (VitD, HDL and BMI); M_4_ : Model -4 (VitD, TG and BMI).

|  | represents odds ratio < 1; |
| --- | --- |
|  | represents odds ratio > 1; |
|  | Variable not in the model. |

**Supplementary table 6:** Multivariate association of PD with vitamin B12 and lipids as scale variables.

| **Outcomes →** | **Prediabetes** | | | |
| --- | --- | --- | --- | --- |
| **Exposures ↓** | **M_1_** | **M_2_** | **M_3_** | **M_4_** |
| VitB12 | NS | NS | NS | NS |
| CHOL | 2.287  (1.234 – 4.238)  0.009 |  |  |  |
| LDL |  | 1.466  (1.116 – 1.925)  0.006 |  |  |
| HDL |  |  | NS |  |
| TG |  |  |  | NS |
| BMI | NS | NS | NS | NS |
| age | NS | NS | NS | NS |

Values represents odds ratios with 95% confidence intervals and exact p value (< 0.05); NS: Statistically not significant.

M_1_ : Model-1 (VitB12, CHOL, BMI and age);

M_2_ : Model-2 (VitB12, LDL, BMI and age);

M_3_ : Model-3 (VitB12, HDL, BMI and age);

M_4_ : Model-4 (VitaB12, TG, BMI and age).

|  | represents odds ratio < 1; |  |
| --- | --- | --- |
|  | represents odds ratio > 1; |  |
|  | Variable not in the model. |  |

**Supplementary table 7:** Multivariate association of PD with folate and lipids as scale variables.

| **Outcomes →** | **Prediabetes** | | | |
| --- | --- | --- | --- | --- |
| **Exposures ↓** | **M_1_** | **M_2_** | **M_3_** | **M_4_** |
| Folate | NS | NS | NS | NS |
| CHOL | 2.406  (1.307 – 4.430)  0.005 |  |  |  |
| LDL |  | 1.503  (1.148 – 1.969)  0.003 |  |  |
| HDL |  |  | NS |  |
| TG |  |  |  | NS |
| BMI | NS | NS | NS | NS |
| age | NS | NS | NS | NS |

Values represents odds ratios with 95% confidence intervals and exact p value (< 0.05); NS: Statistically not significant.

M_1_ : Model-1 (Folate, CHOL, BMI and age);

M_2_ : Model-2 (Folate, LDL, BMI and age);

M_3_ : Model-3 (Folate, HDL, BMI and age);

M_4_ : Model-4 (Folate, TG, BMI and age).

|  | represents odds ratio < 1; |
| --- | --- |
|  | represents odds ratio > 1; |
|  | Variable not in the model. |

**Supplementary table 8:** Multivariate association of PD with vitamin D and lipids as scale variables.

| **Outcomes →** | **Prediabetes** | | | |
| --- | --- | --- | --- | --- |
| **Exposures ↓** | **M_1_** | **M_2_** | **M_3_** | **M_4_** |
| VitD | NS | NS | NS | NS |
| CHOL | 2.434  (1.321 – 4.485)  0.004 |  |  |  |
| LDL |  | 1.509  (1.152 – 1.976)  0.003 |  |  |
| HDL |  |  | NS |  |
| TG |  |  |  | NS |
| BMI | NS | NS | NS | NS |
| age | NS | NS | NS | NS |

Values represents odds ratios with 95% confidence intervals and exact p value (< 0.05); NS: Statistically not significant.

M_1_ : Model-1 (VitD, CHOL, BMI and age);

M_2_ : Model-2 (VitD, LDL, BMI and age);

M_3_ : Model-3 (VitD, HDL, BMI and age);

M_4_ : Model-4 (VitD, TG, BMI and age).

|  | represents odds ratio < 1; |
| --- | --- |
|  | represents odds ratio > 1; |
|  | Variable not in the model. |
